# Supplementary figures and images for: Quantifying Live Microbial Load in Human Saliva Samples over Time Reveals Stable Composition and Dynamic Load
Source: mSystems. 2021 Feb 16;6(1):e01182-20. doi: 10.1128/mSystems.01182-20 (PMC8561659; doi:10.1128/mSystems.01182-20)

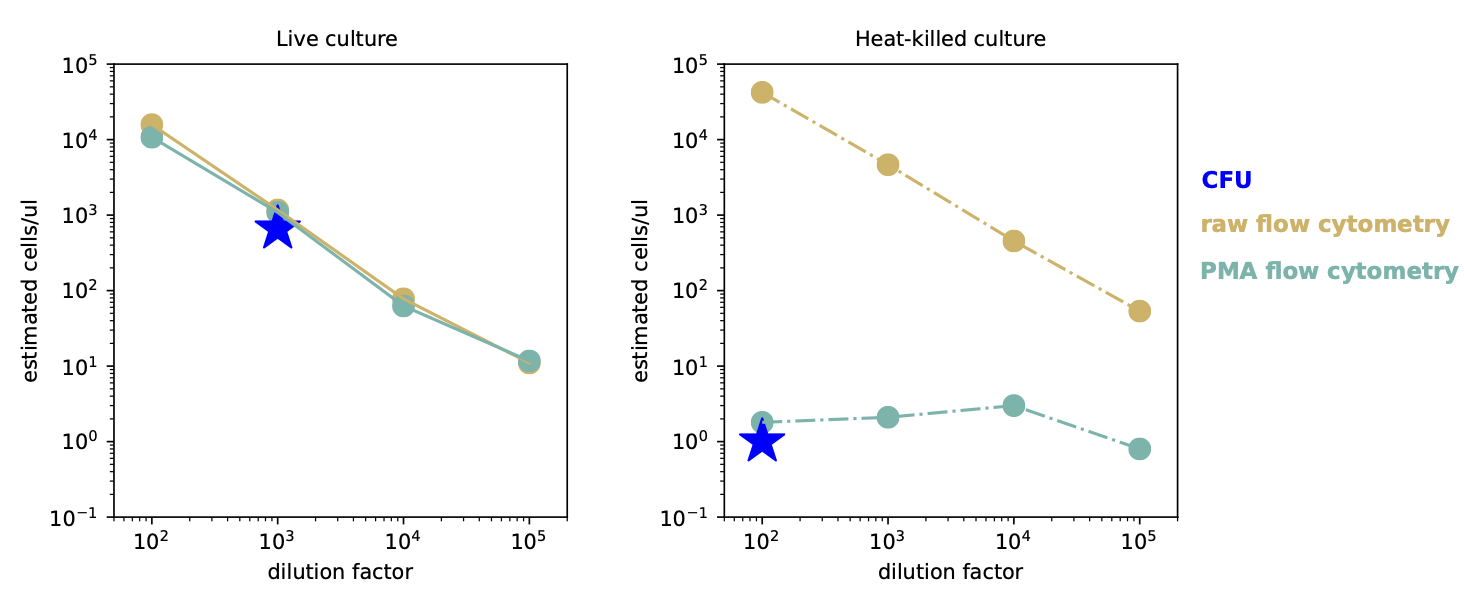

Supplement: FIG S1 [file msystems.01182-20-sf001.tif]

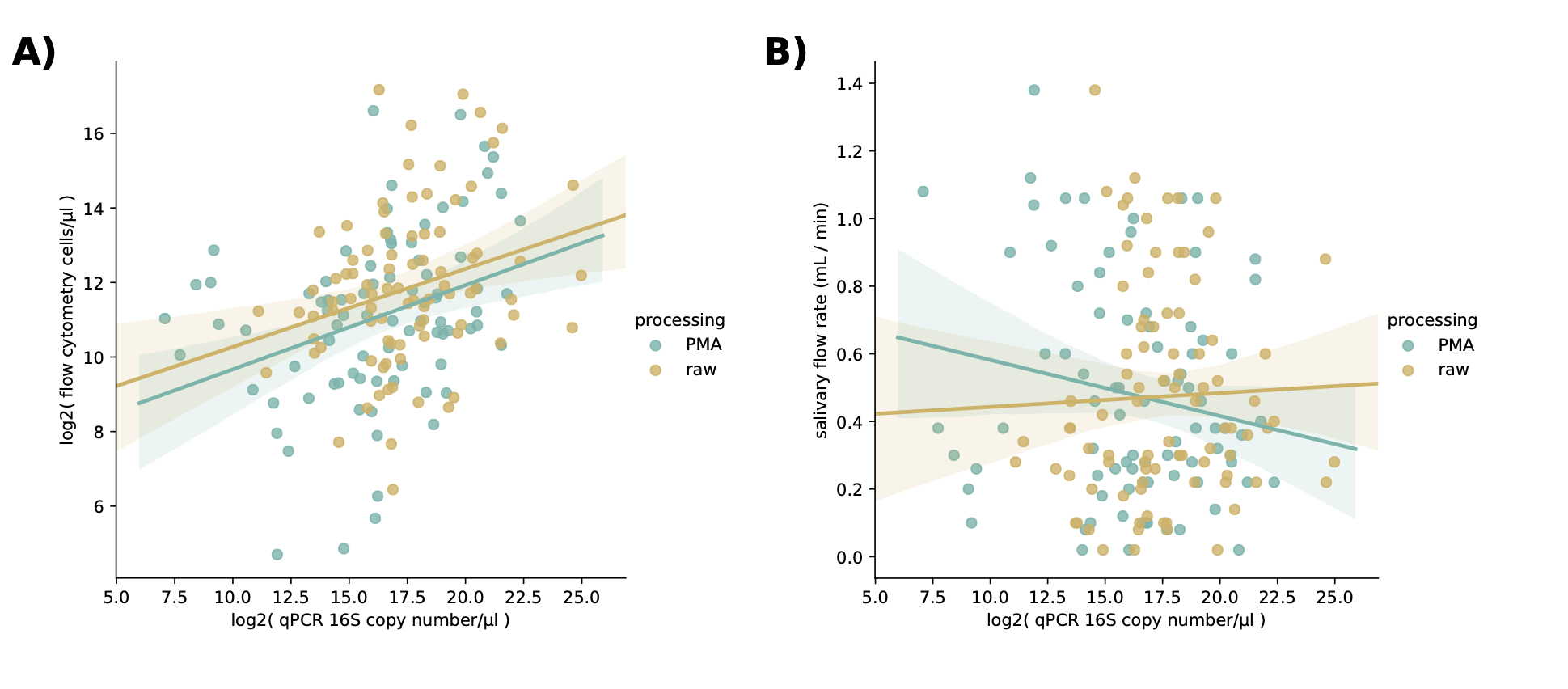

Supplement: FIG S2 [file msystems.01182-20-sf002.jpg]

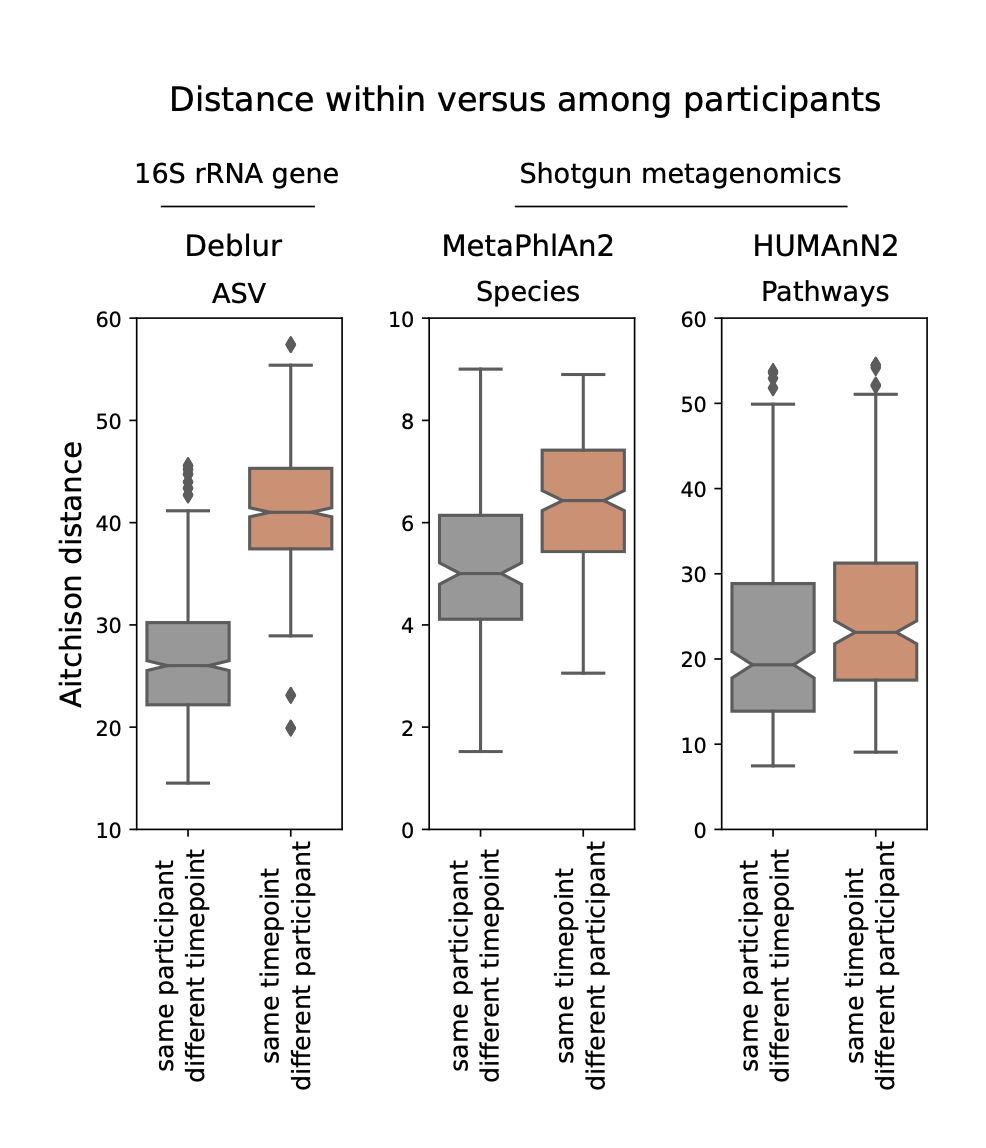

Supplement: FIG S3 [file msystems.01182-20-sf003.jpg]

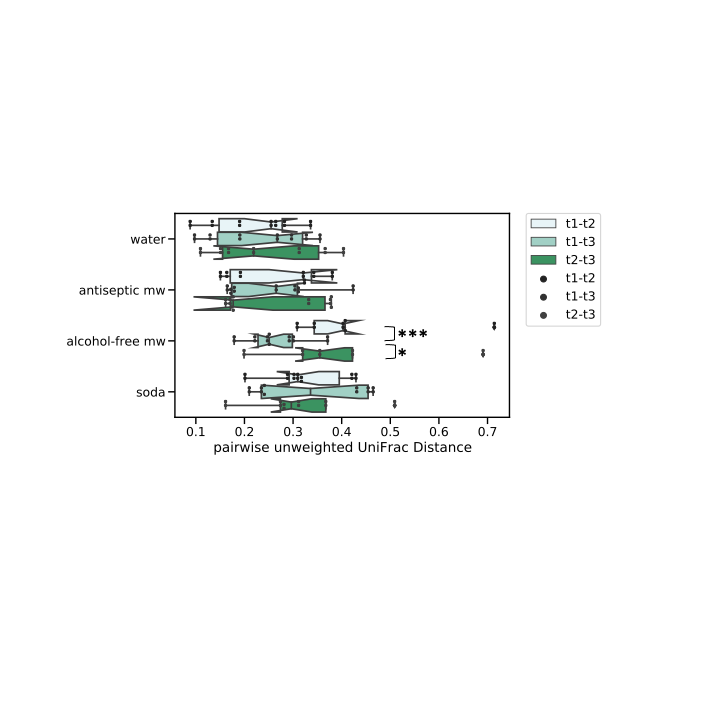

Supplement: FIG S4 [file msystems.01182-20-sf004.tif]

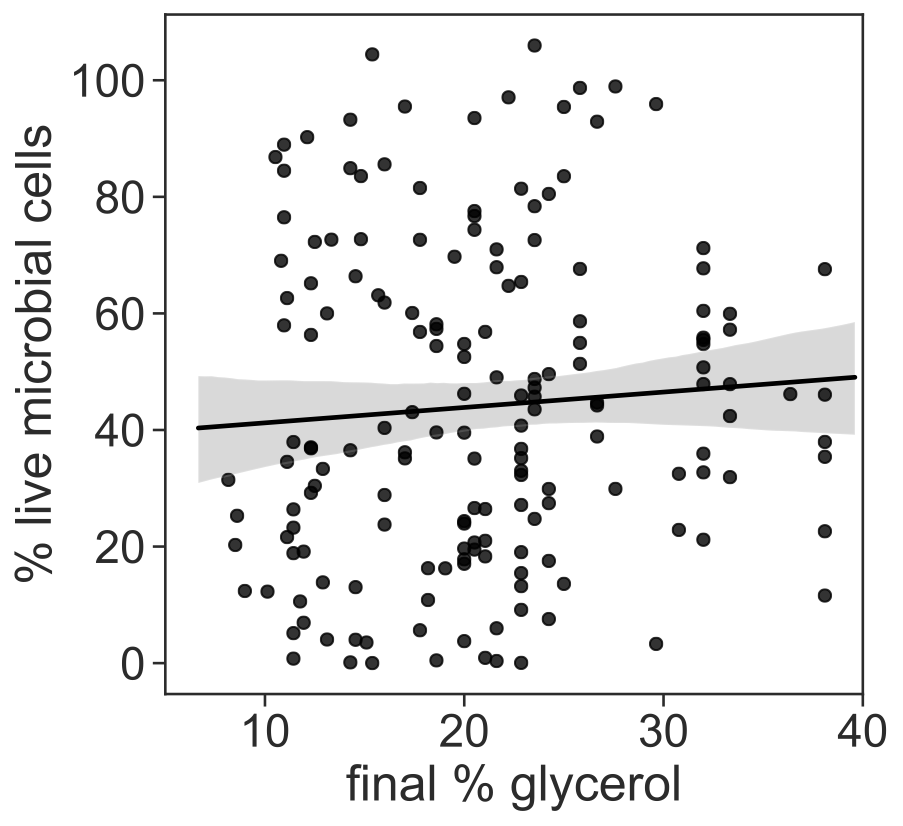

Supplement: FIG S5 [file msystems.01182-20-sf005.tif]

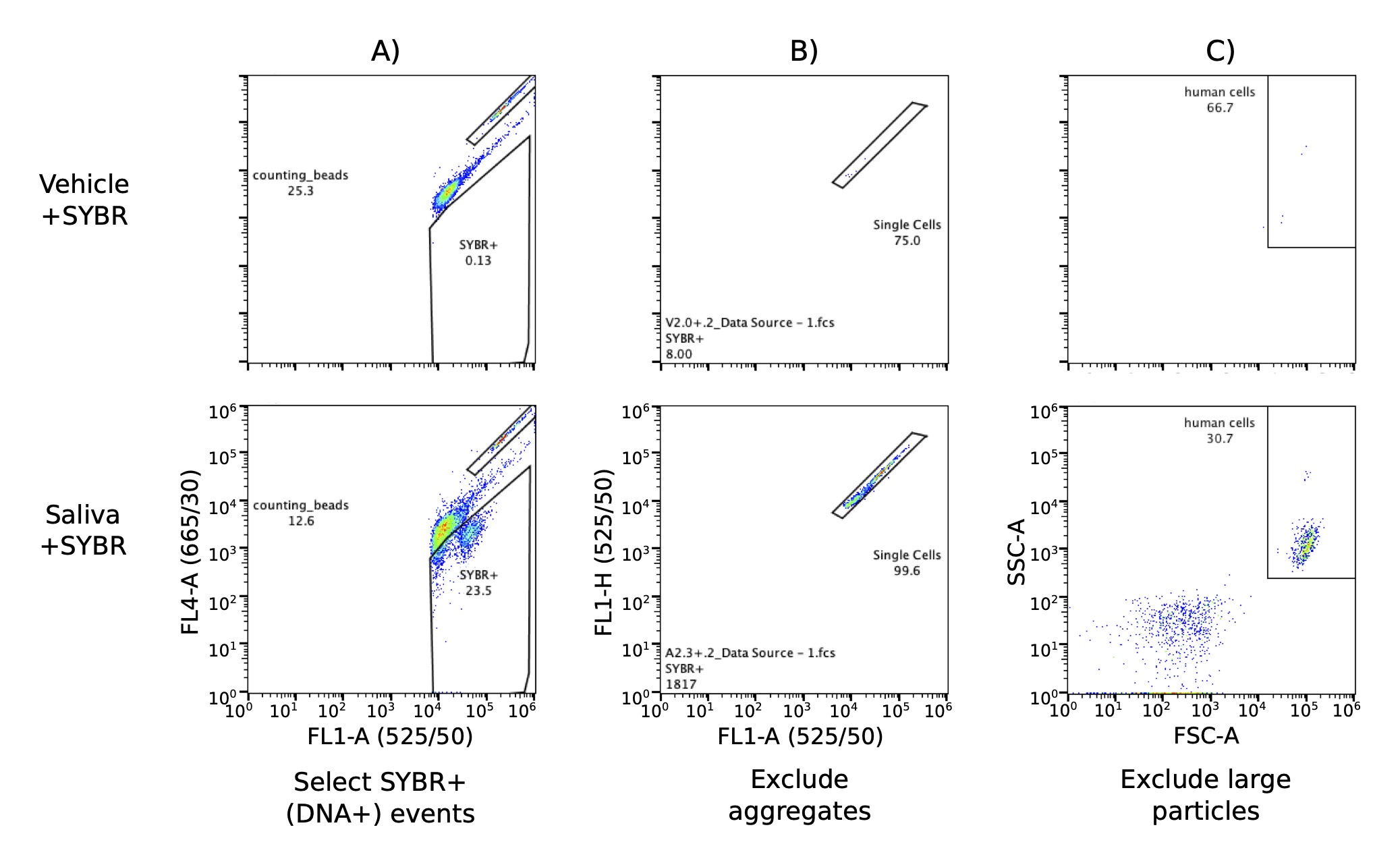

Supplement: FIG S6 [file msystems.01182-20-sf006.jpg]

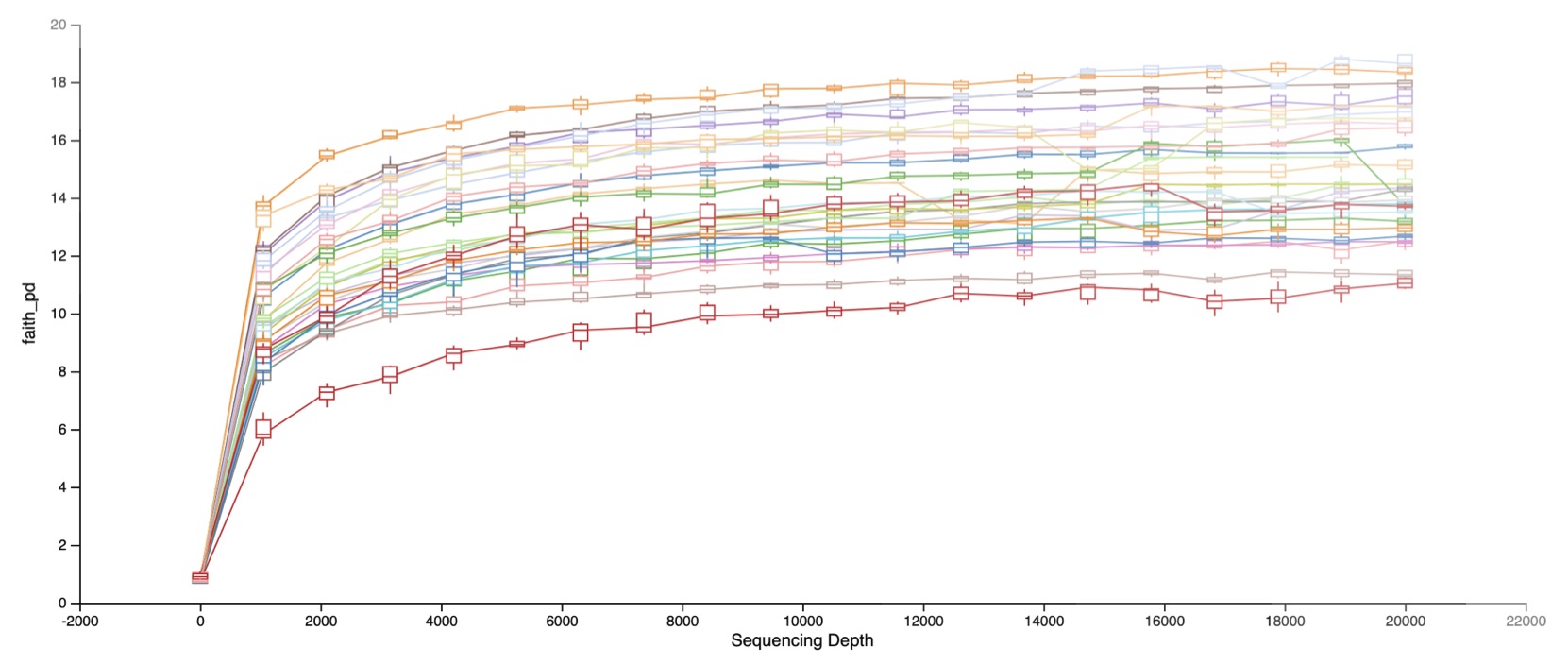

Supplement: FIG S7 [file msystems.01182-20-sf007.jpg]
